# *Bacillus velezensis* as a model for plant-associated beneficial bacilli

**DOI:** 10.1128/jb.00519-25

**Published:** 2026-02-13

**Authors:** Rainer Borriss, Xuewen Gao, Ben Fan

**Affiliations:** 1 Institute of Biology, Humboldt University 9373 , Berlin, Germany; 2 Department of Plant Pathology, Nanjing Agricultural University 214176, Nanjing, China; 3 Co-Innovation Center for Sustainable Forestry in Southern China, College of Forestry and Grassland, Nanjing Forestry University 74584, Nanjing, China; Dartmouth College Geisel School of Medicine, Hanover, New Hampshire, USA

**Keywords:** *Bacillus velezensis*, FZB42 strain, rhizobacteria, giant gene clusters, non-ribosomal synthesis, lipopeptides, polyketides, bacterial volatiles, induced systemic resistance

## Abstract

The *Bacillus* strain GB03, the first representative of a group of plant growth-promoting rhizobacteria, now designated *Bacillus velezensis*, was isolated as *Bacillus subtilis* A13 around 50 years ago from a wheat field in Australia. With the advent of genome sequencing, FZB42, another example of the same taxonomic group of plant-associated gram-positive bacteria, was sequenced in 2007. FZB42 and other *B. velezensis* strains devote a much higher proportion of their whole genomic capacity than the model *B. subtilis* to the synthesis of secondary metabolites with antimicrobial action. This review summarizes the history of discovery and agricultural use, as well as the impressive accumulation of our knowledge base about the mutualistic interactions of *B. velezensis* with plants obtained during the last two decades.

## HISTORICAL BACKGROUND: EARLY INVESTIGATIONS

Today, plant-associated bacilli are widely applied in the biocontrol of plant diseases and promoting plant growth. *Bacillus* strains are advantageous due to their ability to form resistant endospores and are well suited for large-scale fermentation (1).

**Fig 1:**
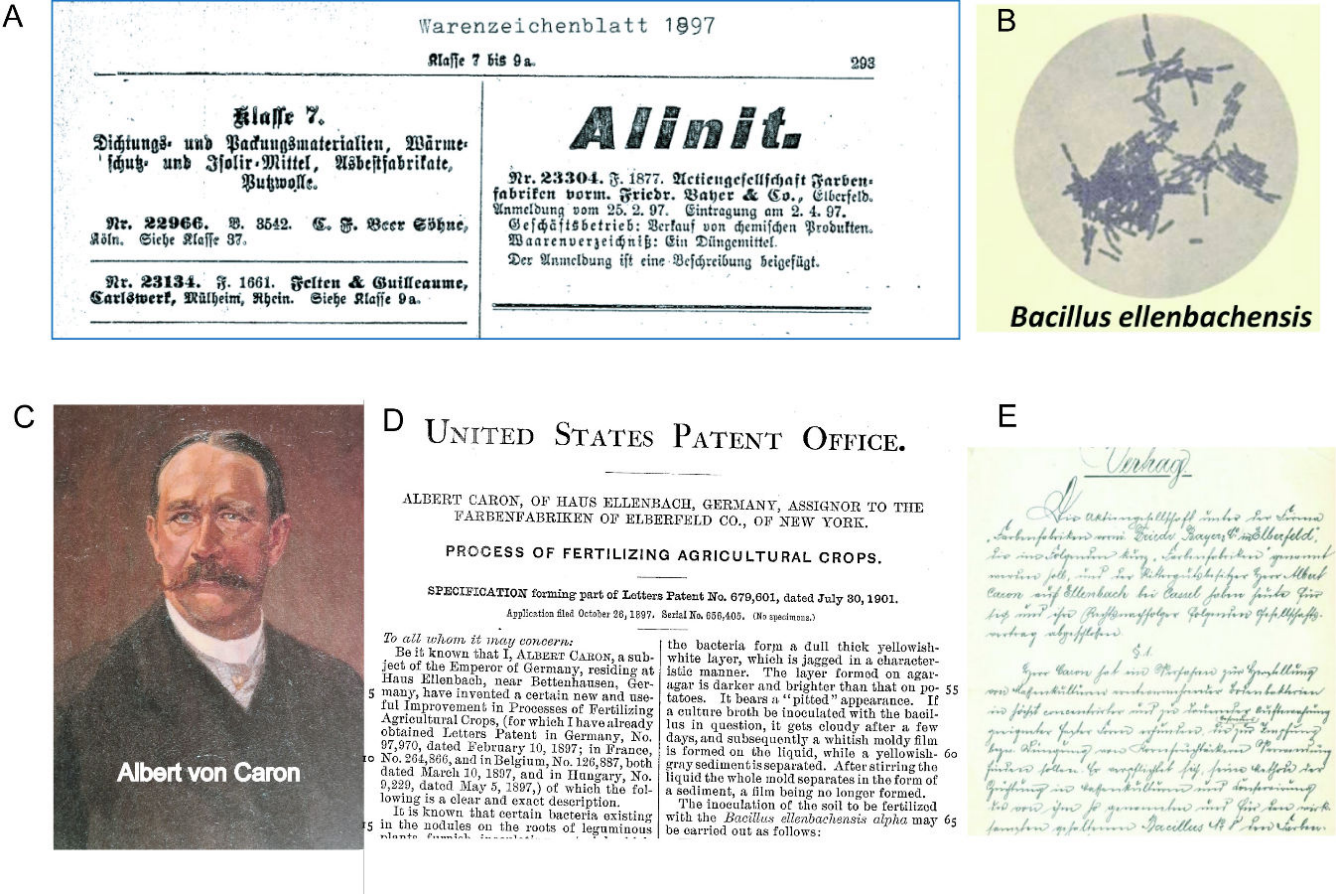
Left: the landowner Albert Caron (1853−1933) filed a patent for the “meadow-Bacillus No. 8,” later designated as *B. ellenbachensis*, which increases harvest yield in cereals. (**A**) Caron’s invention was commercialized as Alinit in 1897 by Farbenfabriken vorm. Friedrich Bayer & Co. (**B**) Micrograph of *B. ellenbachensis*. (**C**) Albert von Caron. (**D**) Caron’s US patent “Process of Fertilizing Agricultural Crops” filed in 1901. (**E**) Contract between Farbenfabriken, Elberfeld, and Albert Caron, Ellenbach. Single file used in this figure is courtesy of Mr. Michael Pohlenz, Bayer Business Services GmbH, Information Center, Corporate History Archives, Leverkusen, Germany.

Early observations about the beneficial effect on plant growth accomplished by plant-associated soil bacteria, nowadays designated as plant growth-promoting rhizobacteria (PGPR), can be traced back to the 19th century (Fig. 1). A bacteriological fertilizer for seed treatment of cereal crops was marketed in 1897 by “Farbenfabriken vormals Friedrich Bayer & Co.” in Elberfeld, now Wuppertal-Elberfeld/Germany. The product “Alinit” is based on the pioneering work of the German landowner Albert von Caron (1853–1933) on his estate in Ellenbach near Kassel (2, 3). Caron isolated the Alinit bacterium *Bacillus ellenbachensis* (previously known as *Bacillus* a and *Bacillus* b) from meadow soil and claimed that he had obtained a considerable increase in harvest yield by applying this bacterium. His studies were supported by Dr. Alfred Koch (1858–1922), the first director of the Institute of Agricultural Bacteriology, Göttingen, Germany (4).

Unfortunately, *B. ellenbachensis* was never validly published. According to later experiments, the Alinit bacterium seems to be related to either *Bacillus megaterium*, nowadays *Priestia megaterium* (5), or *Bacillus subtilis* (6). To date, no DNA sequences of *B. ellenbachensis* are available, and the exact taxonomic position of *B. ellenbachensis* remains elusive. Later*,* as cheap and efficient chemical fertilizers and pesticides became available, the early microbial formulations, such as Alinit, were no longer applied in agriculture for a long time period. However, today, as the negative impact of overusing agrochemicals on the environment is increasingly noticed, the use of environmentally friendly biologicals in agriculture is increasingly reconsidered.

One of the first attempts started with the isolation of *B. subtilis* A13 from wheat-field soil in Glen-Osmond, South Australia (7). Broadbent et al. (8) confirmed the original finding of Caron that inoculating soils with bacilli promotes seedlings’ germination and enhances crop growth. They found the effect to be plant-specific and generally greater under low-nutrient conditions. Unlike in Rhizobia, the contribution of direct mechanisms such as nitrogen fixation to the observed plant growth-promoting effects could be excluded. A13 was successfully used for the treatment of peanut seeds and cotton and was shown to enhance harvest yield in field experiments (9). Around a century after Caron and the Bayer “Farbenfabriken” developed the biological fertilizer “Alinit,” Kloepper and the Texas-based seed company Gustafson developed a novel bioproduct supporting plant health, “Kodiak,” based on a plant-associated *Bacillus* strain, which has been renamed by Gustafson as “GB03” (10). Today, Kodiak is distributed as a biological fungicide by Bayer CropScience with durable GB03 spores as the active ingredient (11). In the following years, an increasing number of plant-associated *Bacillus* strains with the ability to promote plant growth and suppress plant pathogens were isolated from plant rhizosphere, roots, and leaves. In many cases, they have proven to be effective as biocontrol agents, biofertilizers, or biostimulants when applied in sustainable agriculture.

## *BACILLUS VELEZENSIS,* THE KEY SPECIES FOR PLANT GROWTH PROMOTION AND BIOCONTROL

In recent years, gram-positive, plant-associated bacilli emerged as a group of efficient PGPR that colonize plant roots and produce various antimicrobial compounds to protect plants from pathogens (12). In 2018, the genus *Bacillus* consisted of more than 280 validly published species (13) and was considered a taxonomic and phylogenetic anomaly. A unifying feature of the numerous species within the genus *Bacillus* is their ability to form endospores under aerobic conditions, but, with the advent of genome sequencing, they were shown to exhibit extensive polyphyly. As a consequence, modern taxonomy distinguishes 17 monophyletic clades, in addition to the genus *Bacillus*. Today, the genus *Bacillus* is restricted to members of two independent clades: the Subtilis clade (containing the type species *B. subtilis*) and the Cereus clade (containing human-pathogenic *Bacillus anthracis* and *Bacillus cereus*) (5).

The first strains later classified as being *B. velezensis* were originally described as *B. subtilis* (7, 12, 14). After the refinement of taxonomic analysis, especially by whole-genome analysis (15), it became obvious that a special group of low-GC, gram-positive plant-beneficial rhizobacteria, closely related to *Bacillus amyloliquefaciens*, represents a distinct ecotype and was proposed to be considered as a subspecies “*plantarum*” (16). Later, it was established by comparative genomic analysis that the type strain *B. amyloliquefaciens* subsp. *plantarum* FZB42^T^ was tightly related to *B. velezensis* KCTC13012T, which was first described in 2005 (17). Consequently, due to the priority rule, *B. amyloliquefaciens* subsp. *plantarum* is now reclassified as *B. velezensis* (18). Our extended analysis, based on *rpoB* and *gyrB* gene sequences and whole genome sequences, revealed that the representatives of the free-living soil bacteria *B. amyloliquefaciens* and the plant-associated *Bacillus siamensis* and *B. velezensis* form a conspecific group above species level, which was proposed to be designated as “operational group” *B. amyloliquefaciens* ([19], Table 1). A fourth, more distant related member of the group, *Bacillus nakamurai*, was later added (20). According to modern taxonomy, GB03 and other plant-associated *Bacillus* strains listed in Table 2, originally classified as *B. subtilis,* were redesignated as *B. amyloliquefaciens*, and now as *B. velezensis* (11).

**TABLE 1:** Lineage of *B. velezensis*

Domain	Bacteria	Reference
Kingdom	Bacillati^*a*^	(21)
Phylum	Bacillota (Firmicutes)	
Class	Bacilli^*a*^	(21, 22)
Order	Caryophanales (Bacillales)	(23)
Family	Bacillaceae^*a*^	(24)
Genus	*Bacillus* ^*a*^	(5, 25)
Species complex	*B. subtilis* group	(26, 27)
Operational group	*B. amyloliquefaciens* group	(19, 28)
Species	*B. velezensis* ^*b*^	(17, 18)

^*a*^ Type genus is *Bacillus* Cohn 1872 (Approved Lists 1980).

^*b*^ Type strain is *B. velezensis* CCUG 50740 (17).

**TABLE 2:** Most relevant *B. velezensis* strains used in laboratory and field studies

Strain, accession	Origin	Characteristics	Reference
GB03 “Kodiak” (52)^*a*^	Isolated as *B. subtilis* A13 from lysed mycelium in the soil of a wheat field (Australia).	First *B. velezensis* strain demonstrating plant growth promotion and biocontrol. First commercial biocontrol agent (Kodiak). Whole genome sequence (CP049904.1). Volatiles were identified for the first time as promoting plant growth and eliciting induced systemic resistance.	(7, 11, 29, 30)
QST713 “Serenade” (47)^*a*^	Isolated as *B. subtilis*	Commercialized biofungicide. Whole genome sequence (CP025079.1).	(31, 32)
FZB42 BGSC: 10A6, DSM:2311 “Rhizovital” (176)^*a*^	Isolated as *B. subtilis* from pathogen-infested sugar beet field close to Wanzleben, Magdeburg, Germany	Genetically amenable; first genome sequence (CP009725.2) of gram-positive, plant growth-promoting bacteria; identification of unique gene clusters involved in the synthesis of secondary metabolites (e.g., bacillaene, difficidin, macrolactin, plantazolicin, amylocyclicin, and bacillothiazoles), transposon library. Commercialized as Rhizovital.	(14, 15, 19, 33)
SQR9 (64)^*a*^	Isolated as *B. subtilis* from the rhizosphere of healthy cucumber grown in pathogen-infested field (China)	Genetically amenable; extended transcriptome analysis, effect of maize root exudate. Whole genome sequence (CP006890.1). Research subject for conventional plant-beneficial mechanisms (antimicrobial metabolite production, induced systemic resistance, resource competition, phytohormone production, and novel mechanisms unique to SQR9, such as root development enhancement, nitrogen uptake promotion, and abiotic stress tolerance).	(34–37)

^*a*^ Numbers in parentheses list the number of articles referred to in PubMed (September 2025).

The plant-associated *B. velezensis*, a member of the *B. subtilis* species complex, is widely applied as a powerful biocontrol agent (BCA) and known to produce an array of antagonistic metabolites (38). Since its recognition as a valid species in March 2016, 1,340 publications have been recorded in PubMed. *B. velezensis* is Generally Recognized As Safe (39), which permits its industrial use.

Today, *B. velezensis* is by far the most important bio-source for commercial products used for plant protection (BCA) and plant growth promotion (PGP) in crop plants, but also remains to be the most frequently misidentified species in commercial products (40). The combination of both PGP and BCA traits in *B. velezensis*, along with the inherent shelf stability of *Bacillus* strains (due to the formation of endospores), has made this species extensively utilized in numerous commercial agriculture products (41).

## IMPORTANT STRAINS OF *BACILLUS VELEZENSIS*

Origin and features of important *B. velezensis* isolates used in sustainable agriculture and extensively studied as a model for plant-bacteria interactions are summarized in Table 2.

*B. velezensis* GB03 was originally isolated in 1971 as *B. subtilis* A13 (7), as mentioned above. According to modern taxonomy, GB03 was reclassified as *B. amyloliquefaciens* and later as *B. velezensis*. It has been commercialized as a plant protectant, Kodiak, in 1998 and shown to be efficient against abiotic stress (drought stress and salt stress) in crop plants. Groundbreaking scientific research with GB03 was performed by Joe Kloepper (Auburn University) and Choong-Min Ryu (Auburn University, later KRIBB, Daejeon, South Korea). Bacterial volatile compounds (BVCs) synthesized by GB03 were shown for the first time to promote plant growth and act as elicitors of plant-induced systemic resistance (29). A comprehensive review of GB03 has been published recently (11).

*Bacillus velezensis* FZB42 was isolated from soil obtained from a pathogen-infested sugar beet field in Germany (14). FZB42 was the first gram-positive, plant growth-promoting bacterium, which has been fully sequenced (15). FZB42 is genetically amenable, and numerous gene clusters have been characterized by combining chemical and genetic methods (Table 3 [19]). The commercial biocontrol agent Rhizovital was developed from FZB42 endospores (42).

**TABLE 3:** Biosynthetic gene clusters involved in the synthesis of secondary metabolites with antimicrobial activity, which are invariably conserved in *B. velezensis*

Product	BGC/occurrence in FZB42/reference	Antagonistic action against plant-pathogens and competitors	Induced systemic resistance
Cyclic lipopeptides, non-ribosomal synthesized, Sfp-dependent			
**Surfactin,** NRPS (Type I)^*e*^	BGC0000433.5, 342,619–368,777^*a*^ (43)	*Agrobacterium tumefaciens* (crown gall disease in cherry trees [44])	Induced systemic resistance (ISR) response against *Pseudomonas syringae* infecting *Arabidopsis* (45) ISR responses in perennial ryegrass against *Magnaporthe oryzae* (gray leaf spot [46]) ISR response against *Botrytis cinerea* in *Arabidopsis* (47) ISR response against *Rhizoctonia solani* (lettuce bottom rot [48])
**Bacillomycin D^*c*^/Iturin A^*d*^**, NRPS (Type I)^*e*^ + T1PKS^*f*^	BGC0001090.5, 1,871,179–1,908,429^*a*^^,^^*c*^ (43), BGC0001098.5 (49)	Inhibition of *Fusarium oxysporum* (43)*, Fusarium graminearum* (*Fusarium* head blight in wheat [50])	ISR response against *P. syringae* infecting *Arabidopsis* (45) ISR response against *B. cinerea* infecting *Arabidopsis* (45) ISR response against *R. solani* (lettuce bottom rot [48])
**Fengycin,** NRPS (Type I)^*e*^	BGC0001095, 1,927,940–1,971,947^*a*^ (43)	Inhibition of *F. oxysporum* (43) and *F. graminearum* (51)	ISR response against *P. syringae* infecting *Arabidopsis* (45) ISR response against *R. solani* (lettuce bottom rot [48])
**Bacillibactin**, NRPS (Type I)^*e*^, NRP-metallophore^*e*^	BGC0001185.5, 3,019,050–3,033,995^*a*^ (52)	Inhibition of *Verticillium*, *Fusarium*, *Rhizoctonia*, *Phytophthora* (53) Inhibition of *Pseudomonas aeruginosa* and *Aeromonas veroni* in aquaculture (54),	
Polyketides, ribosomal synthesized, Sfp-dependent			
**Macrolactin H,** T1PKS^*f*^ transAT-PKS^*g*^	BGC0000181.5, 1,391,844–1,450,533 (15, 55, 56)	*A. tumefaciens* (crown gall disease in cherry trees [44]) Inhibition of *B. cereus* (37)	ISR response against *P. syringae* infecting *Arabidopsis* (48)
**Bacillaene,** NRPS (Type I)^*e*^ + T1PKS^*f*^ transAT-PKS^*g*^	BGC0001089.5, 1,700,352–1,776,24^*a*^ (55)	Inhibition of *Bacillus circulans* (37)	ISR response against *B. cinerea* infecting *Arabidopsis* (45)
**Difficidin**, T1PKS^*f*^ transAT-PKS^*g*^	BGC0000176.5 (55, 57)	*Erwinia amylovora* (fire blight disease in orchard trees [57]) *Xanthomonas oryzae* (bacterial blight, bacterial leaf streak of rice [58]) *A. tumefaciens* (crown gall disease in cherry trees [44]) Inhibition of *B. megaterium, B. circulans* (37)	ISR response against *B. cinerea* infecting *Arabidopsis* (45) Enhanced expression of jasmonic acid (JA), ethylene (ET), and salicylic acid pathways (44)
Bacteriocin, ribosomal synthesized			
**Amylocyclicin,** RiPP^*h*^, head_to_tail cyclized	BGC0000616.4, 3,043,470–3,050,619^*a*^^,^^*b*^ (59)	*B. subtilis* and other closely related gram-positive bacteria (59)	
**LCI antimicrobial peptide**	310,859–311,143^*b*^ (60, 61)	*Aeromonas hydrophila* in aquaculture (61)	
Dipeptide, non-ribosomal synthesized, Sfp-independent			
**Bacilysin,** other	BGC0001184.4, 3,588,030–3,601,179^*a*^ (15, 52)	*Erwinia amylovora* (fire blight disease in orchard trees [57]) *X. oryzae* (bacterial blight, bacterial leaf streak in rice [58]) *A. tumefaciens* (crown gall disease in cherry trees [44]) Oomycete (soybean root rot [62])	ISR response against *B. cinerea* infecting *Arabidopsis* (45) Enhanced expression of JA, ET, and salicylic acid pathways (44)

^*a*^ Gene clusters were identified by antiSMASH version 8.0 (63).

^*b*^ Gene clusters were identified by BAGEL version 4 (64).

^*c*^ Related Iturin-like compounds, such as Bacillomycin D, are synthesized by similar biosynthetic gene clusters (BGCs) occurring in *B. velezensis* (65).

^*d*^ Related Iturin-like compounds, such as Iturin A, are synthesized by similar BGCs occurring in *B. velezensis* (65).

^*e*^ Non-ribosomal peptide synthetase.

^*f*^ Type I polyketide synthase.

^*g*^ Trans-AT PKS.

^*h*^ Ribosomally synthesized and post-translationally modified peptide products.

Long-term intensive research has established FZB42 as a model strain for plant-growth-promoting bacilli. Sophisticated genetic tools, such as the Cre-LoxP and CRISPR-Cas9 systems (66), have been applied to generate marker-less mutant strains, elucidating the tripartite interactions between FZB42, plant pathogens, and host plants. A comprehensive review about *B. velezensis* FZB42 has been published in 2018 (38). A total of 177 research articles focusing on FZB42 have been published and recorded in PubMed.

Recent large field trials performed in the Central Highlands in Vietnam revealed that closely related variants of the strain were highly efficient in suppressing phytopathogenic nematodes, oomycetes, and fungi and enhanced harvest yield in pepper and coffee trees (67).

*Bacillus velezensis* SQR9 was isolated from a healthy cucumber plant grown in a heavily pathogen-infested field by a group of researchers at Nanjing Agricultural University led by Ruifu Zhang and Qirong Shen (68). Similar to FZB42, SQR9 is also genetically amenable and able to promote plant growth and control plant pathogens. Significant findings have been obtained with SQR9, which has helped elucidate several mechanisms underlying plant-bacteria interactions and provided novel insights into root colonization processes (34–37).

## BIOCONTROL: SECONDARY METABOLITES AND VOLATILES

Recent studies (40, 67) confirmed that *B. velezensis* strains, due to their rich spectrum of biosynthetic gene clusters (BGCs), are more efficient than representatives of other species in inhibiting plant pathogens, such as pathogenic fungi (*Fusarium* spp., *Rhizoctonia solani*), oomycetes (*Phytophthora nicotianae*), and nematodes (*Meloidogyne* spp.). Nine BGCs and the gene for the synthesis of the antimicrobial LCI peptide are strictly conserved in the genomes of *B. velezensis*. Eight of them are involved in non-ribosomal synthesis of lipopeptides (surfactin, fengycin, iturin-like compounds, and siderophore bacillibactin), polyketides (bacillaene, macrolactin, and difficidin), and the dipeptide antibiotic bacilysin. The biosynthetic macrolactin gene cluster seems to be unique for *B. velezensis* genomes, while the difficidin biosynthetic gene cluster is also present in *B. siamensis*, another member of the *B. amyloliquefaciens* operational group.

**Fig 2:**
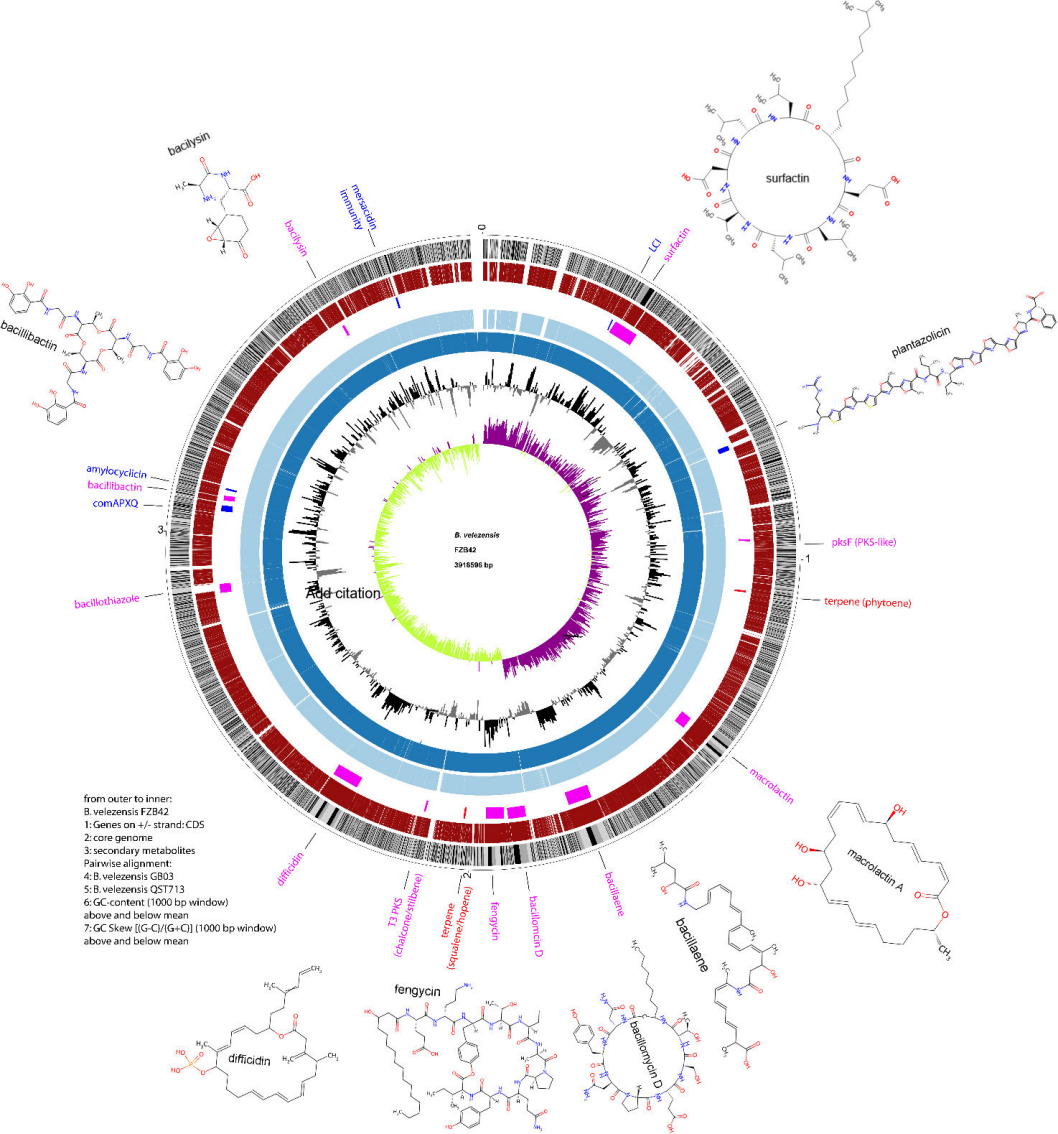
The genome sequence of *B. velezensis* FZB42 contains a multitude of giant gene clusters involved in non-ribosomal synthesis of lipopeptides (surfactin, bacillomycin D, and fengycin), bacillothiazole, the siderophore bacillibactin, and polyketides (macrolactin, bacillaene, and difficidin). Other BGCs present in FZB42 are involved in the synthesis of chalcone/stilbene, terpenes (phytoene and squalene), and bacteriocins (LCI, plantazolicin, amylocyclicin, and the mersacidin immunity proteins). The well-conserved genomes of two other important *B. velezensis* strains (GB03, QST713) are indicated as fourth and fifth inner rings, colored light blue and blue.

By contrast, the biosynthetic bacillaene, fengycin, and surfactin gene clusters can be detected in all members of the *B. subtilis* group. Synthesis of cyclic lipopeptides and polyketides is dependent on 4′-phosphopantetheinyl transferase (Sfp), while non-ribosomal synthesis of the antibacterial dipeptide bacilysin is independent of Sfp. The circular bacteriocin amylocylicin is ribosomally synthesized, modified, and exported by a cluster of six genes (59). The nine BGCs have been first described in the model strain *B. velezensis* FZB42 (Table 3). Other BGCs occasionally occurring in *B. velezensis* are the non-ribosomally synthesized bacillothiazols (BGC0002641.2 [69]), bacillunoic acid (70), and several ribosomally synthesized and post-translationally modified peptides, such as plantazolicin (BGC0000569.5 [71]), subtilin/ericin (BGC0000511.5 [72]), and mersacidin (BGC0000527.5 [73]). In addition to the BGCs generally conserved in *B. velezensis*, the gene clusters involved in the synthesis of bacillothiazole and plantazolicin were also detected in *B. velezensis* FZB42 (Fig. 2).

The cyclic lipoheptapeptide surfactin helps bacteria move on solid surfaces and is abundantly produced during root colonization, thereby eliciting induced systemic resistance against pathogens in plants (74). Secondary metabolites secreted by *B. velezensis* were considered for a long time as responsible for biocontrol of plant pathogens due to their direct antagonistic action (direct antibiosis). However, several of them were found to support plant health indirectly as elicitors of induced systemic resistance (ISR) (see below). Resource competition accomplished by the iron siderophore bacillibactin is another possibility for biocontrol (37). An overview is given in Table 3.

It can be assumed that the expression of antimicrobials synthesized by *B. velezensis* is subject to modulation under the competitive conditions of the plant rhizosphere. Andrić et al. (75) used dual interaction assays to demonstrate that *B. velezensis* mobilizes its whole arsenal of secondary metabolites, such as cyclic lipopeptides, polyketides, the bacillibactin siderophore, the dipeptide bacilysin, and the bacteriocin amylocyclicin upon sensing *Pseudomonas* metabolites. Especially, the synthesis of the polyketide bacillaene was found to be enhanced after sensing the *Pseudomonas* pyochelin siderophore by *B. velezensis* under iron starvation conditions. The authors conclude that this kind of metabolic response after sensing competitors contributes to enhanced fitness and persistence of *B. velezensis* within the highly competitive environment of the plant rhizosphere.

A blend of numerous low-molecular, volatile compounds (BVCs), including biologically active alcohols (2,3-butanediol), hydrocarbons (tridecane), and ketones (acetoin, 2-nonanone), is emitted by *B. velezensis* GB03 and IN937a (76). Besides other functions, such as plant growth promotion, BVCs were found to control plant diseases caused by bacterial (*Pseudomonas syringae* and *Pseudomonas carotovora*) and fungal pathogens (*Botrytis cinerea* [77]). It is generally assumed that eliciting ISR is the main mechanism by which BVCs act against plant pathogens. Direct growth inhibition effects by BVCs were obtained by using relatively high volatile concentrations, not occurring in natural environments, whereas significant ISR effects were obtained at extremely low concentrations of the volatiles, occurring in natural environments (78).

## COMPETITION WITH OTHER SOIL ORGANISMS

In their natural environment, such as the rhizosphere, bacteria can either cooperate or compete. Kin discrimination allows rhizobacteria to decide whether they collaborate or compete with each other. Cooperation is performed in closely related bacteria recognized as kin, while bacteria recognized as non-kin were attacked as competitors (79). Kin selection is considered an important mechanism to stabilize population cooperation, but the molecular tools to distinguish kin from non-kin bacteria are far from clear. Experiments performed with *B. velezensis* SQR9, equipped with heterologous flagellar filaments, point out that polymorphism detected in flagella within the species is decisive in cooperation between *B. velezensis* strains. In this way, *B. velezensis* might reshape the *Bacillus* community composition by increasing the number of cooperative strains in the rhizosphere (80).

The type VII secretion system (T7SS) of *B. velezensis* is involved in the contact-dependent inhibition of competing neighboring bacteria. The LXG-domain containing protein is expressed by *B. velezensis* in the presence of root exudates and suppresses the growth of competing *Bacillus* cells inhabiting the same ecological niche (81).

*B. velezensis* is well equipped with chemical weapons that directly or indirectly act against its competitors in colonizing plant roots (Table 3). The unique antibacterial bacillunoic acid toxin is non-ribosomally synthesized by a type 1 fatty acid synthase and type a 1 PKS by *B. velezensis* SQR9. Both enzymes are encoded on a genomic island and seem to be acquired by horizontal gene transfer. Bacillunoic acids lyse closely related *Bacillus* strains competing with SQR9 in the plant rhizosphere, such as *B. velezensis* FZB42. An ABC transporter, responsible for self-immunity of SQR9 but not present in FZB42, was also encoded on the genomic island (70).

Pot and field experiments pointed out that colonization of lettuce roots by *B. velezensis* FZB42 has no durable effect on the composition of the lettuce bacteriome, despite the bacterial community showing a clear temporal shift immediately after inoculation, according to T-RFLP analysis. This is in contrast to the durable shift observed after artificial inoculation with *R. solani*, the causal agent of bottom rot in lettuce (82). A more comprehensive study using 16S rRNA amplicon sequencing revealed that γ-proteobacteria, especially representatives of the genus *Pseudomonas,* dominate the core lettuce microbiome, consisting of phyllosphere and rhizosphere. However, the diversity structure of γ-proteobacteria changed after infecting lettuce plants with the fungal pathogen *R. solani*. Notably, simultaneous inoculation with FZB42 counteracts this effect, suggesting a less-known mechanism of biocontrol (83). Applying metagenome sequencing, analysis of the rhizosphere bacterial community affected by the tripartite system consisting of lettuce plants, the pathogenic fungus *R. solani*, and FZB42 was further refined. The most abundant phyla present in samples taken in a time period from inoculation with FZB42 until 5 weeks were Proteobacteria, Actinobacteria, Bacteroidetes, and Firmicutes and represented at least 95% of all sequences. No obvious differences in the microbiome composition in samples inoculated with FZB42 and the untreated control were detected after 2 and 5 weeks, corroborating that the presence of FZB42 has no major impact on the composition of rhizosphere microbiome (84).

## PLANT-BACTERIA INTERACTIONS (1): ELICITING ISR

As early as 2004, Kloepper, Ryu, and Zhang (85) summarized that eliciting ISR, earlier described for fluorescent *Pseudomonas* strains, is an important mechanism for biocontrol used by PGPR bacilli. A precondition for detecting ISR is to ensure spatial distance between the bacterium triggering ISR, normally applied belowground in the rhizosphere, and the target pathogen residing aboveground on plant leaves. In an alternative test system (I-plates), volatiles emitted by *B. velezensis* (former *B. amyloliquefaciens* IN937a and *B. subtilis* GB03) were shown to reduce disease severity in plant leaves infected with *Erwinia amylovora*. Increased expression of the gene marker PDF1.2 was detected, indicating that the ethylene pathway for eliciting ISR was used (29).

By contrast to pathogens, which elicit systemic-acquired resistance, SAR, mutualistic rhizobacteria, such as fluorescent pseudomonads and *B. velezensis,* trigger mainly SA-independent pathways (85). However, exceptions from this rule have been reported (see Table 2). Therefore, it seems to be appropriate to consider SAR elicited by pathogens as a special variant of ISR, which can also be elicited by PGPR, such as *B. velezensis* (85). In the presence of FZB42 wild-type cells, the JA/ET-ISR in lettuce plants is accompanied by increased expression of the marker PDF1.2 defensin. In the simultaneous presence of both antagonists, *B. velezensis* and the plant pathogen *Rhizoctonia solani*, PDF1.2 expression was dramatically increased (48), suggesting a synergistic activation of the JA/ET pathways.

It has been found that the concentration of most secondary metabolites synthesized by plant-associated *B. velezensis in planta* under hydroponic conditions is surprisingly low, suggesting that direct antagonistic effects against plant pathogens might be limited (86). Alternatively, cyclic lipopeptides can trigger plant defense pathways against plant pathogens at low concentrations (87). The production of lipopeptides by FZB42 in the lettuce rhizosphere was followed in an axenic *in situ* system. Mass-spectrometric analyses (UHPLC-qToF-MS) revealed that surfactin, fengycin, and bacillomycin were synthesized. In the presence of the lettuce pathogen *Rhizoctonia solani,* the synthesis of surfactin and bacillomycin D was enhanced (85). Further experiments corroborated that in addition to the cyclic lipopeptides, polyketides, bacilysin, exopolysaccharides, and volatiles contribute to ISR response in plants after infection with plant pathogens. The ethylene (ET), jasmonic acid (JA), and/or salicylic acid (SA) signal pathways are activated during ISR response of different *B. velezensis* strains (45).

The compound 2,3-butanediol controls different pathogens by inducing plant defense reactions, such as the insect pest *Spodoptera littoralis,* the bacterial pathogen *Xanthomonas axonopodas* (bacterial spot disease), the fungal pathogen *B. cinerea* (78), and viral diseases (cucumber mosaic virus, CMV, and tobacco mosaic virus, TMV). The stereoisomers 2R,3R-butanediol and 2R,3S-butanediol were found to be efficient in greenhouse and field trials against CMV and TMV, thereby inducing the expression of the SA, JA, and ET pathways in pepper plants (88). Experiments performed with *Arabidopsis* seedlings infected with *B. cinerea* revealed that biocontrol of the fungal pathogen is mainly due to an indirect mechanism in which BVCs, emitted by GB03, elicit ISR as indicated by the expression of the genes involved in JA- and SA-dependent pathways (78).

The BVCs acetoin and 2,3-butanediol emitted by *B. velezensis* FZB42, but not lipopeptides and polyketides, have been shown to activate the abscisic acid and SA-regulated pathways to induce stomatal closure in *Arabidopsis thaliana* and *Nicotiana benthamiana*, thereby hindering the pathogen *Phytophthora nicotianae* from entering into the inner leaf tissues. The BVCs function either via root absorption or volatilization to restrict stomatal apertures, but root absorption was more efficient. Besides stomatal closure, accumulation of hydrogen peroxide and nitric oxide was stimulated (89).

*B. velezensis* SQR9 elicits ISR in maize infected with *Gibberella* stalk rot, thereby boosting H_2_O_2_ production in cotyledons and callose deposition in leaves. SA and JA signaling pathways are involved in ISR. Ca^2+^ signaling, indicated by the expression of related *CDPK* genes, underlines the complexity of the ISR response elicited by *B. velezensis* (34).

## PLANT-BACTERIA INTERACTIONS (2): FIRST STEPS OF PLANT COLONIZATION

A precondition for successful colonization is the outgrowth of the dormant endospores to active vegetative cells. It has been shown that germination of *B. velezensis* SQR9 is rapidly achieved in the vicinity of plant roots by several amino acids and sugars present in root exudate (90). Germination receptors, GerA, GerB, and GerK, are involved in recognizing different components of the root exudate (91).

**Fig 3:**
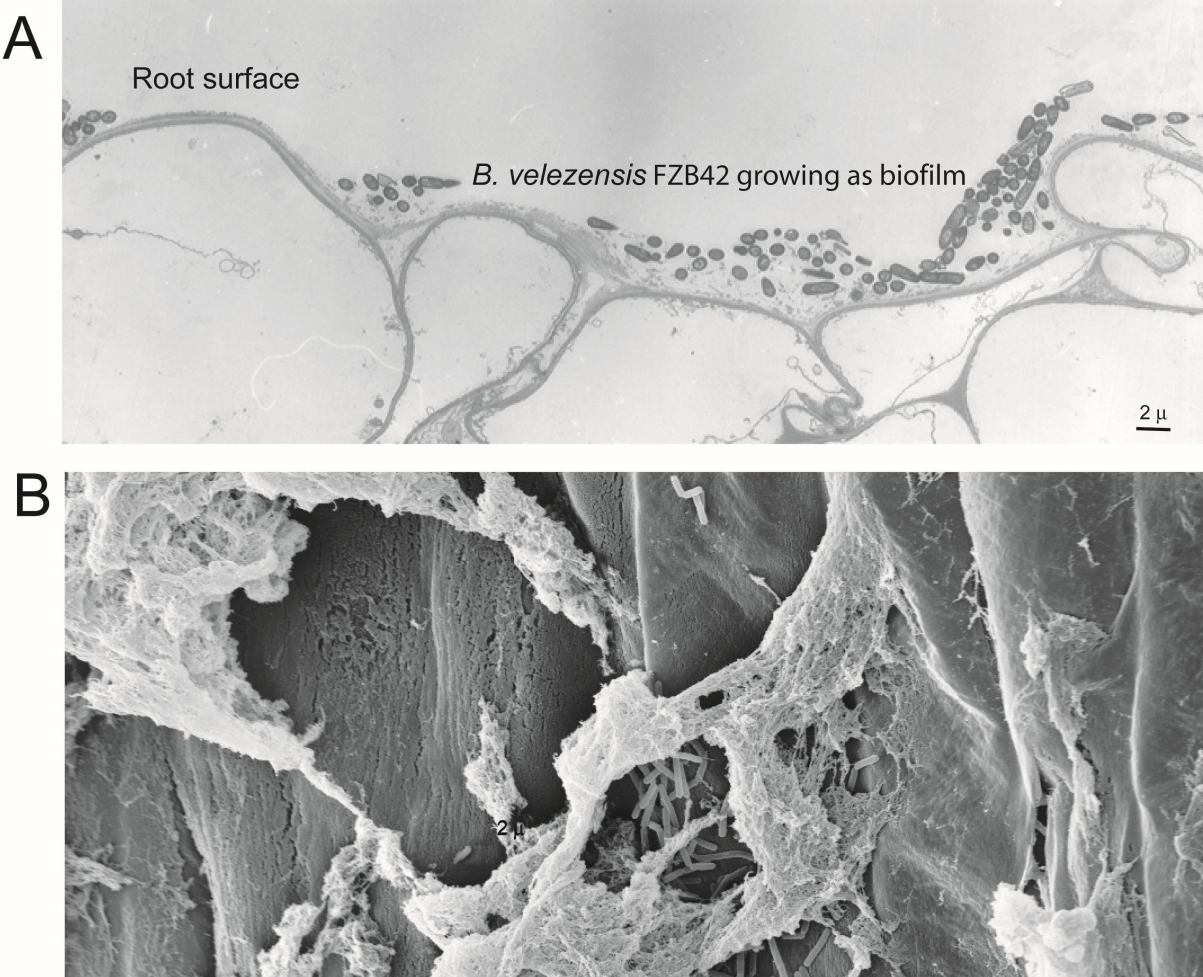
*Bacillus velezensis* FZB42 grows as a biofilm when colonizing maize root surfaces. Images were taken 6 days after bacterial inoculation. (**A**) Transmission electron microscopy of a cross-section taken around 5 cm from the root base. (**B**) Root surface covered with FZB42 biofilm, scanning electron microscopy. Pictures courtesy of Dr. Wilfried Bleiss, Humboldt University, Berlin.

Essential steps for colonizing plant surfaces (Fig. 3), such as roots and leaves, include active movement to target sites (swarming and chemotaxis). *B. velezensis* possesses the necessary genes for swarming, including the *swr* genes and the *fla-che* operon genes encoding flagellar components. As a prerequisite for successful colonization, rhizobacteria are attracted by organic acids, amino acids, and sugars present in root exudates (92). McpA and McpC were identified as the key chemoreceptors for root exudate (93). The ligand-binding domain of McpA directly binds 13 diverse ligands in different ways (94).

An important precondition for the colonization of plant roots by rhizobacteria is their adhesion to plant surfaces. Collagen-like proteins of FZB42 cells were shown to be responsible for auto-aggregation and adherence to the surface of abiotic material and *Arabidopsis* roots. Inactivation of the *clp* genes disrupts biofilm formation (95). ClpB and ClpC proteins, characterized by a distinctive Gly-X-Thr repeating amino acid sequence also occurring in animal collagens, are associated with flagella (96). Other proteins with potential adhesin function, such as the flagellar protein FliD, were identified in SQR9 (97).

Rhizosphere competence of *B. velezensis* is connected with its ability to persist on plant surfaces and withstand plant defense responses triggered by the presence of the beneficial plant-associated bacteria (98). Like other plant-associated bacteria, *B. velezensis* serves as a target of the plant immune system. A first filter for entering the root surface is a basal defense system, which is mediated by plant pattern-recognition receptors sensing microbe-associated molecular patterns (MAMPs), such as bacterial flagellin, elongation factor EF-Tu, bacterial lipopeptides, peptidoglycan, and fungal chitin, common in bacteria and fungi (99). Early MAMP-induced plant defense responses are an efflux of calcium ions and an oxidative burst (local accumulation of reactive oxygen species, ROS). While plant immunity is well understood in its response to pathogens, the interactions between plants and root-colonizing PGPR are an active area of research (100). *B. velezensis* has developed efficient strategies to overcome plant immunity. An oxidative burst in cucumber plants, stimulated during colonization with *B. velezensis,* was induced by flagellin Flg22, yet the rhizobacterium withstood the high oxygen stress. It was demonstrated that the enhanced ROS tolerance of *B. velezensis* was dependent on ResE, a component of the ResD-ResE signal transduction system, which is essential for both aerobic and anaerobic respiration (101). The *nfrA* gene product is involved in rhizosphere competence, protecting bacterial cells against the oxidative stress caused by the plant response during colonization (33). The presence of plant pathogens enhances root colonization by the beneficial bacterium (102).

PGPR FZB42 was shown to synthesize auxin (103) and stimulate lateral root (LR) formation together with biomass accumulation in several plants (104). In an intriguing study, Tzipilevich et al. (100) revealed that the plant hormone auxin (indole-3-acetic acid, IAA) has a direct impact on plant colonization by *B. velezensis*. The FZB42 *ysnE* mutant, impaired in the synthesis of IAA (103), failed to colonize the root as efficiently as the wild type, suggesting that IAA is necessary for *B. velezensis* to survive and grow on the root. Callose deposition, increased ROS production, and expression of immune-related promoters indicated that colonization by *B. velezensis* elicits an early immune response in plant roots. The existence of a feedback loop between ROS production, triggered by the plant immune system, and bacterial IAA production was experimentally confirmed. After triggering ROS production by root-colonizing bacteria, bacterial IAA synthesis is elicited in turn by ROS. As a consequence, IAA supports further root colonization by beneficial bacteria, which protects the plants from their enemies, suggesting a win-win situation for both partners.

The type VII secretion system is involved in promoting root colonization by *B. velezensi*s SQR9 under iron-limited conditions. It was shown that the bacterial T7SS cargo protein YukE is exported and then inserted into the root cell membrane using the bacterial T7SS transport apparatus. The resulting pore in the plant membrane causes iron leakage. The increase of iron in the rhizosphere favors bacterial colonization, ultimately resulting in plant growth promotion by SQR9. The decrease in root iron content was temporary and did not affect plant growth. Besides the known iron acquisition via bacillibactin siderophores, the utilization of iron from plant root cells represents a novel and interesting strategy during root colonization of plants by beneficial rhizobacteria (36).

## PLANT-BACTERIA INTERACTIONS (3): BIOFILM FORMATION ON PLANT SURFACES

Biofilm formation enables *B. velezensis* to persist and thrive in the environment (Fig. 3).

Structural components of *B. velezensis* biofilms include exopolysaccharides encoded by the *epsA-O* operon, γ-polyglutamic acid encoded by *capABC*, and secreted amyloid fiber proteins TasA and TapA encoded by the *tasA-sipW-tapA* operon. The wall teichoic acids are also required for biofilm formation (105). Collectively, these components allow *B. velezensis* to form highly structured, thick, and dense biofilms.

Environmental factors affect the formation of biofilms. In a low-oxygen environment, biofilm formation is enhanced. The ResDE two-component system is involved in sensing the environmental signal and governing biofilm formation (106). Another protein involved in biofilm formation is FtsE, which regulates this complex process, possibly through interaction with the autolysin CwlO and the Spo0A pathway in a not completely understood way (107). The abilities to swarm and form biofilms confer better fitness to the *B. velezensis* bacteria, allowing it to outcompete pathogens and effectively colonize and persist in the target environment (32).

Genes involved in multicellular behavior and biofilm formation were screened from a transposon library of FZB42. DegU, part of the DegS-DegU two-component system, was identified as an activator of genes involved in biofilm formation, and the *degU* mutant was impaired in root colonization (33). Deletion of DegQ and DegU reduced surfactin production and biofilm formation in *B. velezensis* (108). Transcriptional master regulator Spo0A and the repressors AbrB and SinR govern the expression of structural components of the biofilm matrix in *B. velezensis,* similar to that in the closely related *B. subtilis* (37, 109), and it can be assumed that similar regulatory networks exist in both species.

The signal molecule surfactin was reported as being essential for forming biofilms in *B. subtilis* (110) and *B. velezensis* (111). It has been proposed that surfactin accomplishes biofilm formation by regulating the expression of AbrB, DegS-DegU, and SinI-SinR (112). However, it should be noted here that Thérien et al. (74) found that surfactin production is not essential for root-associated biofilm development of *Bacillus subtilis,* suggesting that surfactin-related biocontrol and plant growth promotion are independent from biofilm formation.

Similar to *B. subtilis* 168, *B. velezensis* SQR9 biofilm consists of different subpopulations, which perform specialized functions: extracellular matrix (ECM) producer and cheater-like ECM non-producers. ECM cells also synthesize the “cannibalism” toxin bacillunoic acids (70), to which ECM-nonproducers are sensitive. These functional differences are largely regulated by Spo0A-P (113).

A novel small regulatory RNA named PhoS was shown to regulate biofilm formation in *B. velezensis* FZB42 and *B. subtilis* (114). Initially identified in a genome-wide screen of small non-coding RNAs and validated by Northern blot, PhoS was mapped in the 3′ untranslated region (UTR) of the *phoPR* genes (115). Similar to the phytase *phyC* gene in the environmental *B. velezensis* FZB45 (116), PhoS belongs to the phosphate starvation-inducible PhoPR regulon (117). By contrast to the other regulators of the Pho Regulon, which are all proteins, PhoS represents a different regulator type. Its expression is stimulated by the transcription response regulator PhoP. *Vice versa,* PhoS enhances the translation of PhoP by interacting with the 5′ UTR of *phoP* mRNA. PhoS contributes to biofilm formation, at least partly, by enhancing PhoP expression, which governs the biosynthesis of teichuronic acid under phosphate limitation. The identification of PhoS as part of the complex regulatory network governing phosphate metabolism also has implications for phytate degradation by environmental *B. velezensis* strains such as FZB45, which enhances plant nutrition by mobilizing accessible nutrients in the rhizosphere under phosphate-limiting conditions (118). PhoS was also shown to act in *B. subtilis* (114).

**Fig 4:**
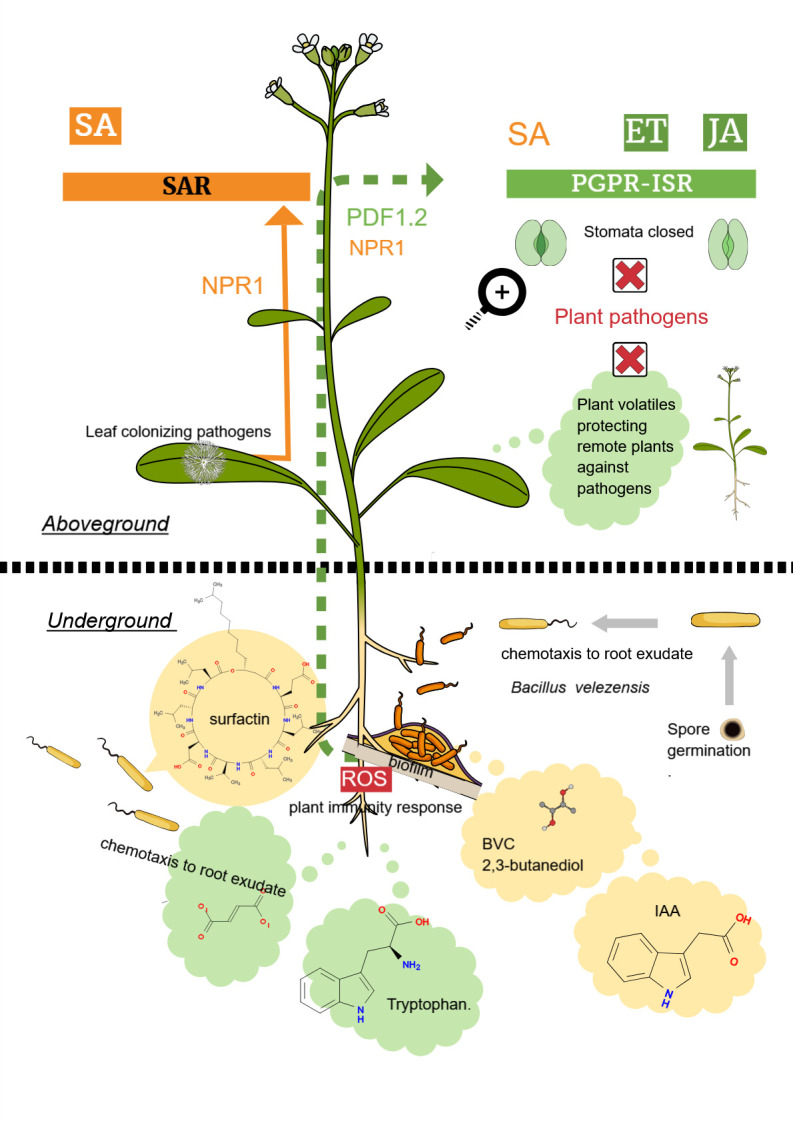
Simplified scheme, illustrating the interactions between plants and *B. velezensis*. After the outgrowth of *B. velezensis* endospores to vegetative cells, chemotaxis driven by plant root exudate guides the bacteria toward plant roots. During root attachment, MAMPs trigger plant immune response, and bacteria need to evade plant defense reactions, such as the production of reactive oxygen species and NO. Bacterial IAA, synthesized from plant root secreted tryptophan, counteracts the plant immune response and ROS toxicity. In a feedback loop, ROS stimulates IAA synthesis and enhances root colonization. *B. velezensis* biofilms, formed on plant surfaces, emit BVCs and produce surfactin, which both elicit induced systemic resistance but can also act directly against plant pathogens. Plant signaling pathways involved in eliciting induced systemic resistance are accomplished by the phytohormones jasmonic acid and ethylene, while salicylic acid is involved in signaling systemic acquired resistance induced by plant pathogens, but SA can also be involved in eliciting PGPR-ISR by beneficial bacteria. Protection against pathogens includes different mechanisms, such as stomatal closure and signal transfer to neighboring plants via gaseous compounds emitted from plant leaves. For more explanations, see the text.

The interactions in the tripartite system—consisting of plants in their natural environment, the beneficial rhizobacteria (*B. velezensis*), and plant pathogens colonizing plant rhizosphere, and aboveground plant surfaces are summarized as a simplified scheme in Fig. 4.

## PLANT-BACTERIA INTERACTIONS (4): PLANT GROWTH PROMOTION AND FURTHER PLANT BENEFITS

*B. velezensis* promotes plant growth by various mechanisms, such as producing the plant growth hormone IAA, promoting root development, emitting volatile compounds, enhancing phosphate and nitrogen uptake, resulting in an overall enhancement of crop and harvest yield (37).

Phytohormones play crucial roles in plant growth and development, including cell division, elongation, and differentiation. Their synthesis is not restricted to plants. Plant beneficial rhizobacteria such as *B. velezensis* are known to promote plant growth and root development by synthesizing the phytohormone indole-3-acetic acid via tryptophan-dependent pathways (103). Unfortunately, the routes for IAA synthesis in *B. velezensis* are not completely elucidated. It was proposed that the products of the *patB*, *yclC*, and *dhaS* genes catalyze IAA synthesis via the indole-3-pyruvic acid (IPyA) pathway. The main intermediate, IPyA, might be decarboxylated to indole-3-acetaldehyde and finally converted to IAA by the *dhaS* gene product (119). Alternatively, IAA synthesis might be mainly accomplished by alternative routes catalyzed by the *ysnE* gene product. *B. velezensis* mutants deficient in YsnE, the key enzyme of IAA synthesis in *B. velezensis*, were found to be impaired in plant growth promotion and in inducing lateral root development (100). Unfortunately, the enzymatic nature of the *ysnE* gene product is still elusive. IAA biosynthesis is probably finalized by the *yhcX* gene product (nitrilase), which converts indole-3-acetonitrile to IAA (120). In addition to the IAA synthesized in plants, exogenous auxin pools supplied by root-associated *B. velezensis* bacteria support the formation of cluster roots under low-phosphate conditions in white lupins. Bacterial cells deficient in YsnE did not trigger cluster root formation (121). Enhanced development of lateral roots has also been attributed to the BVCs emitted by *B. velezensis* (122).

Synthesis of cytokinin in plants, another phytohormone, was shown to be activated by *B. velezensis* as indicated by enhanced expression of cytokinin-related genes ARR5 and LBD3 (123). Cytokinins can stimulate stomatal opening and promote plant growth (124).

BVCs secreted by *B. velezensis* trigger growth promotion by upregulating auxin synthesis and transport in *Arabidopsis* plantlets treated with GB03 (125). Microarray data revealed that bacterial volatiles regulate auxin homeostasis and cell expansion (126). However, BVCs inhibiting plant growth were also identified (127). Crop growth promotion and activation of immune responses were observed under controlled and field conditions. For instance, 2-heptanone and 2-nonanone are involved in plant growth-promoting activity by enhancing photosynthetic capacity, while decane and undecane stimulate plant resistance (128). In addition, BVCs released by *B. velezensis* FZB42 significantly promote primary root elongation and increase LR development in *A. thaliana*, thereby altering root architecture. BVC-mediated modulation of root architecture is closely associated with auxin signaling, particularly its polar transport. Notably, the promotive effects of BVCs on lateral root formation were nearly abolished in auxin signaling mutants (129). Bacillolysin, a secreted protein of *B. velezensis*, also promotes LR development via interaction with plant signaling pathways (130).

*B. velezensis* has not evolved its own system to fix airborne nitrogen, but BVCs emitted by SQR9 promote the expression of uptake systems for nitrate and ammonium in plants (131). Beneficial effects exerted by GB03 emitting BVCs are the increased synthesis of the photosynthetic machinery (132) and the activation of the iron acquisition pathways in plants (133). In the presence of GB03, the solubility of ferric iron was enhanced by acidification of the rhizosphere due to increased exudation of root protons. In addition, iron uptake was increased by enhanced activity of Fe^3+^-chelate reductase and Fe^2+^ transporter.

Enhancing iron (Fe) nutrition is one of the known beneficial features of plant-bacteria interactions. Siderophores solubilize Fe by forming siderophore-Fe complexes, resulting in enhanced Fe bioavailability (134). Recently, it was shown that two rhizobacterial traits, siderophores and biofilms, act together to accumulate Fe on plant roots. Iron accumulated in the matrix of SQR9 biofilms is a direct Fe source for plants, and bacillibactin siderophores released from SQR9 biofilms enhance Fe acquisition in plants (135).

Beneficial rhizobacteria colonize the root surface and are able to secrete enzymes to make potential soil nutrients accessible for plant nutrition. In total, 20%–50% of the total soil phosphorus is estimated to be fixed as phytate and other insoluble polyphosphates. *B. velezensis* FZB45 is able to partially mobilize this hidden nutrient by secretion of extracellular 3-phytase (118), which efficiently degrades the insoluble D/L-Ins (1,2,3,4,5,6)P6 to D/L-Ins (1,2,4,5,6)P5 as the first product of phytate hydrolysis. Plant growth promotion was obtained by applying recombinant 3-phytase to maize seedlings growing under phosphate limitation.

## PROMISING STRATEGIES FOR UTILIZING *B. VELEZENSIS* IN SUSTAINABLE AGRICULTURE

Inconsistent performance under field conditions is still the main problem in utilizing beneficial microbes, including *B. velezensis*, in sustainable agriculture (136). In order to overcome this problem, different promising approaches, such as engineering single customized strains and SynComs and applying encapsulated secondary metabolites and volatiles, can be applied.

Compared to single strains, inoculating plants with multi-strain communities (SynCom) appears as a promising strategy to stabilize the plant microbiome, promote plant growth, and enhance harvest yield (137). Ideally, syntrophic cooperation between *B. velezensis* and other members of the microbial communities can enhance the positive impact on plant growth (138). However, their effectiveness is often found to be affected by undesired antagonistic interactions within the rhizosphere. In order to avoid this, the use of consortia consisting of *B. velezensis* and compatible microbes with a high degree of cooperativity has been recommended (139). Alternatively, undesired antagonistic interactions between the members of the SynCom consortium can be alleviated by eliminating the responsible genes (37).

Besides *B. velezensis, Pseudomonas* spp. are known for their plant-associated life style and their ability to promote plant growth and health. Both *Pseudomonas* Pf5 and *B. velezensis* could control tomato wilt disease caused by *Rhizoctonia solanacearum* (140, 141). In a recent study (142), we expected that a two-strain community (SynCom), consisting of representatives of both taxa, might be more efficient than single strains due to cooperative effects. However, due to the inhibitory effects of the fluorescent *Pseudomonas* Pf5 on *B. velezensis*, no cooperative effect on plant growth and health by the two-strain community was detected. Further experiments revealed that the chlorinated polyketide pyoluteorin, non-ribosomally synthesized by Pf5, negatively affects the functionality of the SynCom by inhibiting the growth of *B. velezensis,* including FZB42. By contrast, replacing the wild-type Pf5 strain with a pyoluteorin-deficient mutant strain abolished the growth suppression of *B. velezensis* within the two-strain community. Simultaneously, expression of genes involved in non-ribosomal synthesis of lipopeptides and polyketides, as well as the genes involved in flagellum synthesis, was enhanced. Removal of the pyoluteorin synthesis enabled the *Pseudomonas ΔpltB* Pf5/*B. velezensis* consortium to coexist harmoniously and to be efficient in biofilm formation, root colonization, and plant disease control. Thereby, *ΔpltB* Pf5 and *B. velezensis* coexist within the same root region and form mixed biofilms. The best effect in controlling tomato wilt disease was obtained when *ΔpltB* Pf5 and *B. velezensis* were co-applied. After the removal of the factor hindering cooperative behavior within the synthetic consortium of *B. velezensis* and Pf5, the SynCom became highly efficient in controlling plant pathogens. Moreover, the colonization of *B. velezensis* in the tomato rhizosphere was supported, and the diversity and stability of the tomato microbiome were enhanced.

Although the release of genetically engineered bacteria in the environment is currently restricted by regulatory authorities, the use of customized environmental *B. velezensis* in agriculture is highly desirable, given that their environmental safety is maintained. It has already been shown that selected strains of *B. velezensis* are genetically amenable (66), but convincing examples for their application are still rare (143). FZB42 has been genetically engineered for enhanced synthesis of antimicrobial peptides (144). Recently, a three-plasmid CRISPR-Cas9 platform was established for *B. velezensis* 916. Replacing the native promoters involved in lipopeptide synthesis with strong *Bacillus* promoters yielded a 6- to 10-fold increase in the production of lipopeptides and enhanced efficiency in biocontrol of fungal disease (rice sheath blight and angular leaf spot [145]).

Besides genetic engineering and random mutagenesis, rhizosphere evolution is an alternative breeding method, which has been successfully applied to obtain *B. velezensis* variants with enhanced rhizosphere competence. Strain SQR9 was used for 20 cycles of directed domestication in the pepper rhizosphere *in situ* (146). Strains with enhanced biofilm formation and IAA and siderophore production were isolated. Finally, a strain variant from SQR9 with improved performance in plant growth promotion was selected. Genome sequencing revealed mutations in genes involved in macrolactin synthesis, chromosome segregation, and the ferrichrome ABC transporter FhuC. Confirmation of the beneficial action of the mutant in field trials is still pending. A similar strategy has been previously used to optimize FZB42 with the goal of enhancing its persistence at plant roots (147). Greenhouse cultures of ornamental plant seedlings were inoculated with FZB42 spores and allowed to grow in unsterile soil for 5 months until harvesting. Then, six bacterial clones resembling FZB42 were reisolated from soil attached to harvested plant material. Strain identification was performed with sequence-specific primers. Genome sequencing of one isolate (ABi01) revealed 99.8% identity with the nucleotide sequence of FZB42, but nucleotide exchanges in at least 12 CDS were registered. A superior effect of Abi01 was registered in field trials with potatoes infected with the causative agent of black scurf, *Rhizoctonia solani*.

The existence of a general signaling system between rhizobacteria and plants, accomplished by volatiles, was detected (148). Tomato plants inoculated with GB03 emit volatiles (MIPV, e.g., β-caryophyllene), which are aerially transported to neighboring receiver plants. Specific root exudates secreted in the rhizosphere by emitter and receiver plants contained an increased amount of salicylic acid, which affects the composition of the rhizosphere microbiome. Notably, the root microbiota was found to be similar in plants treated with GB03 and the surrounding uninoculated MIPV receiver plants, indicating that plant volatiles elicited by bacterial colonization of plant roots can synchronize the microbiome of plants growing distant from the site of bacterial inoculation. The authors propose to use MIPVs as an example of volatile-mediated communication for creating a healthy soil microbiome that finally contributes to a more sustainable agriculture. This could be done by optimizing methods for encapsulation of volatiles and determining the effective distance of their emission.

Around 130 years after Albert Caron started his pioneering experiments with the meadow-*Bacillus*, our knowledge base about mutualistic plant-bacteria interactions has dramatically increased. *B. velezensis* has been established as a scientifically important bacterium and a valuable tool, which is increasingly used in single and multi-strain applications in sustainable agriculture. However, despite all the progress, substantial replacement of agrochemicals by efficient biologicals, such as *B. velezensis*, remains a major challenge.
